# A colorectal cancer genome-wide association study in a Spanish cohort identifies two variants associated with colorectal cancer risk at 1p33 and 8p12

**DOI:** 10.1186/1471-2164-14-55

**Published:** 2013-01-26

**Authors:** Ceres Fernandez-Rozadilla, Jean-Baptiste Cazier, Ian P Tomlinson, Luis G Carvajal-Carmona, Claire Palles, María J Lamas, Montserrat Baiget, Luis A López-Fernández, Alejandro Brea-Fernández, Anna Abulí, Luis Bujanda, Juan Clofent, Dolors Gonzalez, Rosa Xicola, Montserrat Andreu, Xavier Bessa, Rodrigo Jover, Xavier Llor, Víctor Moreno, Antoni Castells, Ángel Carracedo, Sergi Castellvi-Bel, Clara Ruiz-Ponte

**Affiliations:** 1 Galician Public Fundation of Genomic Medicine (FPGMX)-Grupo de Medicina Xenómica-Centro de Investigación Biomédica en Red de Enfermedades Raras (CIBERer)-IDIS, Santiago de Compostela, 15706, Spain; 2 Wellcome Trust Centre for Human Genetics, University of Oxford, Oxford, OX3 7BN, UK; 3 NIHR Comprehensive Biomedical Research Centre, University of Oxford, Oxford, OX3 7BN, UK; 4 Department of Biochemistry and Molecular Medicine School of Medicine University of California, Davis, California, USA; 5 Oncology Pharmacy Unit, Complejo Hospitalario Universitario of Santiago (CHUS), Santiago de Compostela, 15706, Spain; 6 Genetics Department, Hospital de Santa Creu I Sant Pau, Barcelona, 08025, Spain; 7 Pharmacogenetics & Pharmacogenomics Laboratory, Servicio de Farmacia, Hospital General Universitario Gregorio Marañón, Instituto de Investigación Sanitaria Gregorio Marañón,, Madrid, 28007, Spain; 8 Gastroenterology Department, Hospital del Mar, Barcelona, 08003, Spain; 9 Department of Gastroenterology, Hospital Clínic, CIBERehd, IDIBAPS, University of Barcelona, Barcelona, 08036, Spain; 10 Gastroenterology Department, Donostia Hospital, CIBERehd, University of the Basque Country, San Sebastián, 20014, Spain; 11 Gastroenterology Department, Hospital do Meixoeiro, Vigo, 36214, Spain; 12 Section of Digestive Diseases, Internal Medicine Department, Hospital Sagunto, Valencia, 46520, Spain; 13 Servicio de Patologia Digestiva, Hospital Sant PAu, Barcelona, 08003, Spain; 14 Section of Digestive Diseases and Nutrition, University of Illinois at Chicago, Chicago, IL, 60607, USA; 15 Gastroenterology Department, Hospital General Universitario de Alicante, Alicante, 03010, Spain; 16 Cancer Prevention and Control Program, Catalan Institute of Oncology (ICO), Bellvitge Biomedical Research Centre (IDIBELL), CIBERESP, Barcelona, 08907, Spain

**Keywords:** GWAS, SNPs, Colorectal cancer, Spanish cohort, 1p33, 8p12

## Abstract

**Background:**

Colorectal cancer (CRC) is a disease of complex aetiology, with much of the expected inherited risk being due to several common low risk variants. Genome-Wide Association Studies (GWAS) have identified 20 CRC risk variants. Nevertheless, these have only been able to explain part of the missing heritability. Moreover, these signals have only been inspected in populations of Northern European origin.

**Results:**

Thus, we followed the same approach in a Spanish cohort of 881 cases and 667 controls. Sixty-four variants at 24 loci were found to be associated with CRC at p-values <10-5. We therefore evaluated the 24 loci in another Spanish replication cohort (1481 cases and 1850 controls). Two of these SNPs, rs12080929 at 1p33 (P_replication_=0.042; P_pooled_=5.523x10^-03^; OR (CI95%)=0.866(0.782-0.959)) and rs11987193 at 8p12 (P_replication_=0.039; P_pooled_=6.985x10^-5^; OR (CI95%)=0.786(0.705-0.878)) were replicated in the second Phase, although they did not reach genome-wide statistical significance.

**Conclusions:**

We have performed the first CRC GWAS in a Southern European population and by these means we were able to identify two new susceptibility variants at 1p33 and 8p12 loci. These two SNPs are located near the *SLC5A9* and *DUSP4* loci, respectively, which could be good functional candidates for the association signals. We therefore believe that these two markers constitute good candidates for CRC susceptibility loci and should be further evaluated in other larger datasets. Moreover, we highlight that were these two SNPs true susceptibility variants, they would constitute a decrease in the CRC missing heritability fraction.

## Background

Even though genetic susceptibility is thought to be responsible for almost 35% of the variation in colorectal cancer (CRC) risk [1], high penetrance mutations in Mendelian predisposition genes, such as *APC*, the mismatch repair (MMR) genes, or *MUTYH* are only able to explain <5% of cases [2]. The recent advances in the field of genetic epidemiology have validated the hypothesis that at least part of that remaining missing susceptibility lies in the form of multiple common low-risk variants, each conferring a modest effect on disease risk.

Genome-wide association studies (GWAS) are one of the most widespread methodologies for the detection of such susceptibility loci. The procedure (in distinction to candidate-gene association studies) offers an untargeted strategy for the detection of new low-penetrance variants, for it does not assume any *a priori* hypothesis on the location of these loci. This advantage has been proved important, since so far this kind of survey has successfully identified 20 variants at 8q24.21, 8q23.3, 10p14, 11q23, 15q13.3, 18q21.1, 14q22.2, 16q22.1, 19q13.1, 20p12.3, 1q41, 3q26.2, 12q13.3, 20q13.33, 6p21, 11q13.3 and Xp22.2 [3-6]. The combined effect of these variants altogether is thought to explain ~7% of the familial cancer risk [7]. Still, there is a high proportion of genetic contribution to CRC risk that has not been identified.

In this study we have undertaken a new screen for CRC susceptibility variants using a GWAS approach on our cohort of 881 CRC cases and 667 controls from the Spanish population. The use of a Southern-European dataset is a novelty that could lead to the identification of new candidate loci, since all of the populations where GWAS analyses have been conducted so far have been of Northern European origin. Although this may provide additional confirmation of the relationship of the 20 described variants to CRC risk in Southern Europe, we must also consider the possibility that there may be differences, at these or other particular loci in the genome, between these populations.

## Materials and methods

### Study populations

Subjects in Phase I were 882 cases and 473 controls ascertained through the EPICOLON II Project and 194 additional controls from the Spanish National DNA bank. The EPICOLON Consortium comprises a prospective, multicentre and population-based epidemiology survey of the incidence and features of CRC in the Spanish population [8,9]. Cases were selected as patients with *de novo* histologically confirmed diagnosis of colorectal adenocarcinoma. Patients with familial adenomatous polyposis, Lynch syndrome or inflammatory bowel disease-related CRC, and cases where patients or family refused to participate in the study were excluded.

Mean age for cases in Phase I was 71.20 years (SD±0.70). Hospital-based controls were recruited through the blood collection unit of each hospital, together with cases. All of the controls were confirmed to have no history of cancer or other neoplasm and no reported family history of CRC. Controls were randomly selected and matched with cases for hospital, sex and age (± 5 years). Population controls from the National DNA bank were also genotyped, to lessen the deficit of controls. They were matched for sex, age (± 10 years) and geographical origin of the sample with the remaining cases. Both cases and controls were of European ancestry and from Spain (stated, when possible, as all four grandparents being Spanish).

Samples in Phase II consisted of 1436 CRC patients and 1780 controls: samples from Hospital Sant Pau were 125 CRC patients from a previously described cohort [10]; the Hospital Gregorio Marañón dataset consisted of 104 CRC patients participating in a pharmagogenetic survey; Catalan Institute of Oncology (ICO) samples were 439 patients who belonged to the Bellvitge Colorectal Cancer Study; the CHUS hospital in Santiago de Compostela was a subsample of 153 participants included in a pharmacogenetic study; 105 CRC cases and 1330 controls came from the Spanish National DNA bank; and 510 CRC cases and 450 DNA controls belonged to the EPICOLON I Project [8]. Of the cases, 60.4% were male and 39.6% female. Controls were matched for gender. Age mean was 69.61 (SD±0.59) for cases and 52.00 (SD±0.58) for controls. Gender and hospital distribution of samples for case and control groups on both Phases is shown on (Additional file 1: Table S1).

DNA was obtained from frozen peripheral blood by standard extraction procedures for all samples. Cases and controls were extracted in mixed batches to avoid bias.

### Ethical standards

The study was approved by the “Comité Ético de Investigación Clínica de Galicia”, and each of the institutional review boards of the participating hospitals. All samples were obtained with written informed consent reviewed by the ethical board of the corresponding hospital, in accordance with the tenets of the Declaration of Helsinki.

### SNP genotyping and QC

Affymetrix array 6.0 (Affymetrix, CA, USA), which includes probes for almost 1M SNP markers, was chosen to obtain genome-wide coverage for Phase I genotyping. Genotype calling for Affymetrix 6.0 was performed with the Birdseed algorithm, included within Birdsuite v1.4 [11]. Samples were organized in 23 batches of 16<n<99 according to hospital of origin for computational purposes. We obtained valid genotypes for 909 622 SNPs by these means. Quality control of the data, performed mainly with PLINK v1.07 [12], included the removal of both SNPs and samples with genotyping success rates <99% (N=5984 for SNPs and N=0 for samples) and samples with discordant gender between clinical recorded data and Affymetrix-asigned sex (N=7). Hardy-Weinberg equilibrium (HWE) was evaluated and markers with P_HWE_<1x10^-4^ in controls were removed from further analyses (N=6984). SNPs with MAFs below 0.05 (N=221 799) were also eliminated due to low power to detect true signals and to avoid unnecessary noise. Finally, differential missingness between cases and controls was also accounted for by excluding markers with p-values below 1x10^-4^ (N=137). This test compares genotyping error rates for the affected *vs.* unaffected groups in order to avoid an increase in false positive findings due to this bias. Finally, SNPs with poor clustering were also excluded after visualisation with Evoker (briefly, associated SNPs at a selected threshold were selected for comparison of the two intensity channels against each other to manually check the proper assignment of the genotype-calling algorithm) [13]. A total of 674 718 SNPs remained after this filtering.

**Figure 1:**
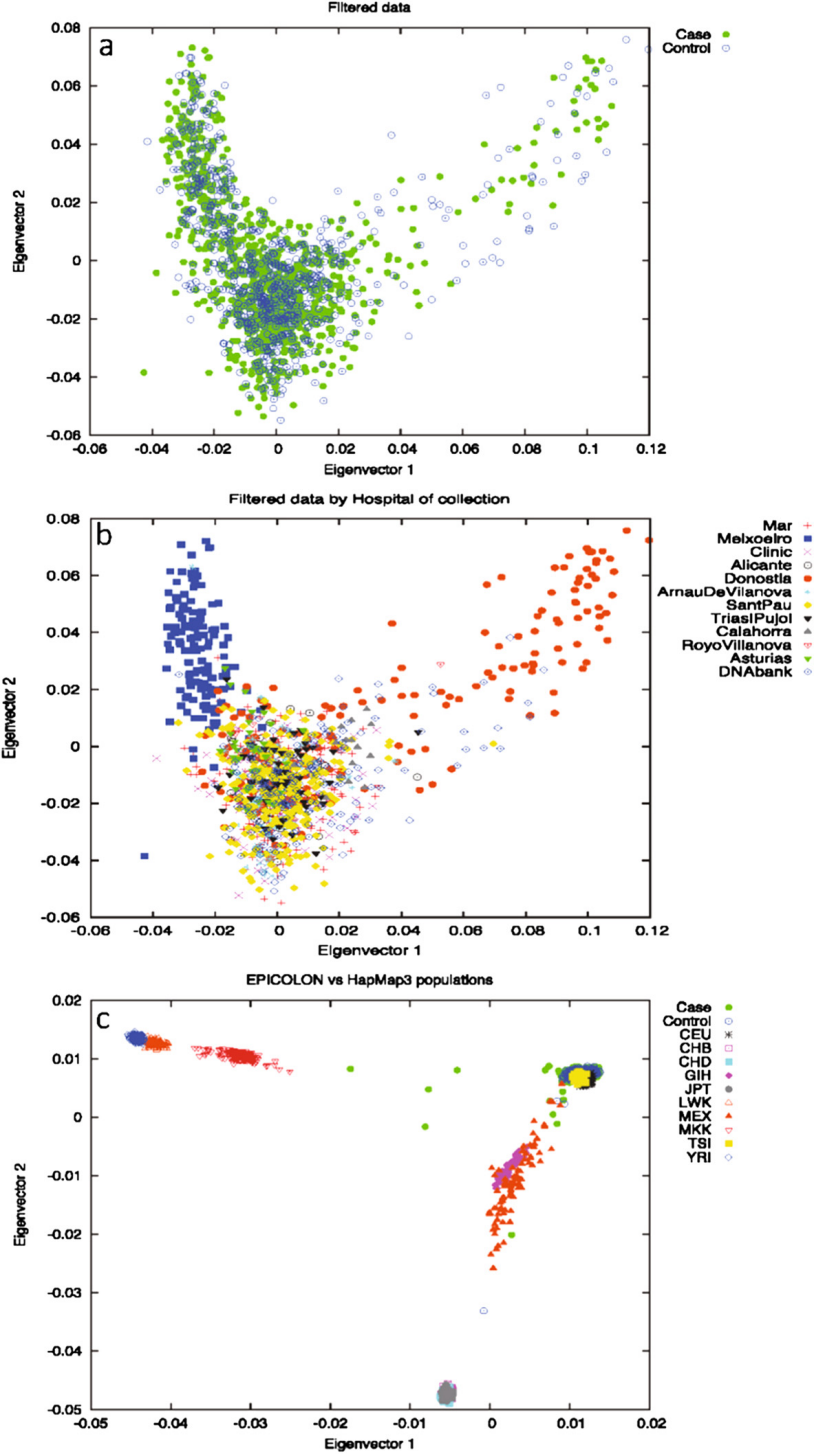
**PCA analysis on the EPICOLON cohort. (a)** filtered data by case/control; **(b)** filtered data by hospital of origin; **(c)** EPICOLON and HapMap3 populations. Significant differences may be seen in section b with Meixoeiro and Donosti hospitals deviating from the main cloud

To address the possibility of underlying population stratification, Principal Component Analysis (PCA) on a subset of 98 986 randomly chosen independent SNPs (pairwise r^2^<0.1) was also performed on the full Phase I cohort using the EIGENSOFT *smartpca* software [14]. Long-range LD regions, as described by Price *et al.*[15], were also removed from this analysis. Outliers, taken as samples spread on principal components 1 and 2 were removed from subsequent analyses, since they deviated from the main cloud. No evidence was found of population differences between cases and controls for the first 10 components of the PCA analysis, as stated in the Tracy-Widsom test (Figure 1a). Other potentially confounding variables, such as markers typed using the Nsp or Sty restriction endonucleases, hospital of collection, genotyping plate, or geographical origin of the samples were also checked for as sources for stratification (data not shown). All results were concordant with the original assumption of a single originating population except for hospital of origin. When considered as a confounding variable, the EPICOLON cohort clustered into three separate subgroups: samples from the Donostia hospital (VAS dataset), the only collection centre for the Basque Country region (North of Spain), samples from the Meixoeiro hospital (GAL dataset), the single collection point in Galicia (NW Spain), and all others (REST dataset) (Figure 1b). An additional PCA comprising the full Phase I cohort and the HapMap3 populations (all ancestries) was also performed to illustrate the clustering of these populations (Figure 1c) [16]. EPICOLON II samples that clustered away from the European end of the plot (showing evidence of non-European ancestry) were excluded from further analyses.

The final case dataset comprised 1477 samples (848 cases and 629 controls). The total count per subgroup was 167 for VAS, 366 for GAL and 944 for REST.

Genotyping in Phase II was conducted by Sequenom MassARRAY technology (Sequenom Inc. San Diego, CA, USA). rs7087402 at 10q23.31 could not be included in the analyses for genotyping design reasons. Quality control was performed with PLINK similarly to Phase I. Genotyping for both Phases was performed at the Santiago de Compostela node of the Spanish Genotyping Centre.

### Statistical analysis

Association analysis was assessed as a 1°-of-freedom χ^2^ allelic test for each of the three subgroups independently for Phase I, and for second Phase replication, with PLINK [12]. The adequacy of the distribution of p-values was evaluated using quantile-quantile (Q-Q) plots of test statistics. Meta-analysis was also conducted using PLINK in Phase I. The method is based on a Mantel-Haenszel approach for data pooling. Cochran´s Q statistic and the I^2^ heterogeneity index were also estimated to account for inter-population heterogeneity between groups, which was defined as I^2^>75% [17,18]. For markers above this threshold, a random-effects model was considered, whereas fixed-effect results were otherwise reported. Risks (odds ratios, ORs) with 95% confidence intervals (CIs) associated with each marker were then estimated assuming the appropriate model. Phase II analyses were adjusted by age, given the mean differences between the case and control populations for these cohorts. Pooled analysis was performed by logistic regression, considering genotyping Phase and population subgroup as covariates. Associations by phenotype (age at diagnosis, MSI status, tumour location, presence of previous adenomas, family history of CRC and sex) were examined by logistic regression in case-only analyses for the two associated SNPs (Additional file 1: Table S2). Additional statistical calculations and plots were performed using R [19].

### Imputation

Imputation between the two recombination hotspots encompassing each of the 24 loci that showed evidence of association in Phase I was accomplished with Impute v2 using two reference panels: 1000 Genomes Project (b36) for wide coverage, and HapMap3 (r2 b36) for deep coverage [16,20,21]. Results from the imputation were later tested for association with SNPTEST [20]. Imputation results were filtered by minor allele frequency (MAF) of the markers (SNPs with MAFs<5% were excluded, since the procedure generates genotypes for a high number of rare variants that could give spurious association results), by missing data proportion (set to a 5% max), and the *frequentist-add-proper-info* column of the output. This latter statistic is the ratio of the empirically observed variance of the allele dosage to the expected binomial variance p(1-p) at HWE, where p is the observed allele frequency from HapMap [22]. Optimal values should be within the (0.4-1) range and provide a measure for quality and accuracy of the imputation. Since the proportion of cases and controls deviates significantly from the standard 1:1, we also considered the possibility that the imputed genotype probabilities for each marker were different in both subsets. Since IMPUTE gives back the imputation results as a probability for each genotype, we decided to filter out SNPs for which the probability of two out of the three genotypes was ≥25% (i.e. the genotype in this sample was not clear) in at least 5% of the cases or the controls. This way we can eliminate samples for which genotype imputation has been inaccurate. Imputation results were plotted with the SNAP on-line tool [23].

## Results

### Stratification within the EPICOLON cohort

We observed using PCA that there was a batch effect due to differences by hospital at which sample had been collected, thereby dividing the EPICOLON cohort into three separate subgroups. We thus proceeded on the basis that each cluster - the GAL, VAS and REST case-control groups - was a separate sample set. Association results were then obtained for each of the subpopulations separately and then meta-analysed. Q-Q plots for the subgroups (after QC) showed an improvement in the systematic inflation for the distribution of the association p-values for the GAL, REST and VAS subgroups (Additional file 2: Figures S1A, 1B and 1C. respectively). Lambda genomic factor calculations (1.04192, 1.02323 and 1.02292 for GAL, VAS and REST, respectively) were consistent with no evidences of an increased false discovery rate. Additional file 2: Figure S1D represents the Q-Q plot distribution after meta-analysis of the three subpopulations. Some SNPs still seem to deviate from the expected distribution. These were later discovered to be artefacts of the calling procedure and were further removed with Evoker.

### Association analyses

We found 64 SNPs at 24 genomic loci SNPs associated with CRC risk at P≤10^-4^ (lowest p-value=9,7x10^-8^ for rs11996339 at 8p12) (Table 1). Notably, I^2^ heterogeneity values for the three subgroups (GAL, VAS, REST) were all 0 for these markers, thereby reflecting homogeneity in these associated SNPs.

**Table 1:** Association results for phase I

**LOCUS**	**SNP**	**BP**	**Allele**	**MAF cases**	**MAF controls**	**OR (95% CI)**	**P value**
1p33	rs12080929	48.208.735	C	0.249	0.312	0.731 (0.621-0.860)	9.447E-05
2p25.2	rs4669394	5.541.078	C	0.049	0.087	0.731 (0.622-0.860)	3.113E-05
2p24.1	rs1554267	22.284.451	A	0.457	0.371	1.429 (1.231-1.658)	3.139E-06
3p21.31	rs8180040	47.363.951	A	0.366	0.454	0.695 (0.599-0.806)	1.595E-06
3q12-q13	rs6438550	121.019.507	G	0.038	0.072	0.511 (0.369-0.709)	6.789E-05
5q35.1	rs11740081	172.707.280	A	0.123	0.188	0.604 (0.494-0.741)	6.789E-05
6q16.1	rs12213685	99.288.865	G	0.163	0.106	1.643 (1.313-2.042)	1.136E-05
6q23.1	rs12199765	131.192.418	A	0.268	0.202	1.434 (1.217-1.726)	5.428E-05
8p12	rs11996339	29.386.099	C	0.351	0.448	0.667 (0.574-0.774)	9.697E-08
8p12	rs11987193	29.391.927	A	0.237	0.309	0.691 (0.589-0.818)	9.752E-06
8p12	rs12548021	29.400.381	G	0.378	0.289	1.478 (1.279-1.749)	1.073E-06
8q13.3	rs17788534	72.697.475	C	0.198	0.141	1.509 (1.237-1.841)	7.903E-05
8q22.1	rs3104964	96.664.912	C	0.452	0.367	1.422 (1.225-1.652)	5.201E-06
10p15.1	rs7074607	5.623.371	A	0.151	0.1025	1.556 (1.242-1.949)	8.901E-05
10q23.31	rs7087402	92.760.125	A	0.527	0.4436	1.398 (1.208-1.619)	5.202E-06
12q24.31	rs568489	119.578.624	G	0.478	0.397	1.391 (1.200-1.612)	1.650E-05
13q32.3	rs17196583	99.624.356	A	0.201	0.145	1.488 (1.222-1.812)	7.307E-05
14q31.3	rs7148493	85.094.169	G	0.431	0.362	1.332 (1.147-1.548)	9.823E-05
14q32.12	rs8177528	92.247.404	A	0.407	0.335	1.367 (1.174-1.591)	5.471E-05
15q21.3	rs4644804	52.164.106	A	0.387	0.316	1.368 (1.173-1.596)	5.403E-05
15q25.3	rs16941001	86.249.170	A	0.101	0.059	1.806 (1.361-2.396)	3.704E-05
17p13.2	rs16954697	5.297.637	A	0.150	0.098	1.625 (1.293-2.044)	2.522E-05
17p12	rs9898623	13.255.126	A	0.045	0.085	0.512 (0.378-0.692)	1.625E-05
18p11.22	rs10502376	8.579.765	A	0.426	0.496	0.753 (0.651-0.872)	9.819E-05
18q21.2	rs2958182	51.300.019	A	0.330	0.261	1.392 (1.184-1.636)	9.792E-05
22q12.3	rs956119	34.582.213	G	0.064	0.104	0.591 (0.453-0.770)	9.193E-05

Main features of the SNPs associated in Phase I. Only the top associated SNPs at each of the 24 independent loci is shown. All genomic positions correspond to NCBI36/hg18.

Using imputation, we examined the LD blocks defined by recombination hotspots (as obtained from Haploview in the CEU+TSI HapMap3-r2 populations) around the 64 SNPs for evidence of stronger signals of association. Additional file 1: Table S3 provides a summary of the loci and extent of the imputed regions. This analysis improved the association at 4 of the 24 loci: 1p33 (best SNP rs12060081); 14q31.3 (rs2057115); 15q21.3 (rs7176932); and 22q12.3 (rs17725348) (Additional file 3: Figure S2).

### Replication

The ±1Mb regions flanking all 24 loci with evidence of association in Phase I were examined in the United Kingdom CORGI GWAS cohort through proxy SNP assessment [24]. Only 5 of these locations showed to have some CORGI associated SNP at an established p value threshold of P≤10^-4^. However, LD analysis (in HapMap3-r2 CEU+TSI available data) showed them all to be independent signals (data not shown). Therefore, our attempt to replicate the association signals *in silico* was not successful for any of the variants.

**Figure 2:**
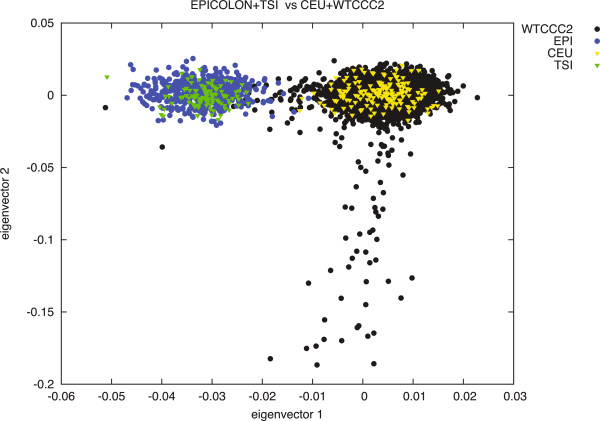
**PCA analysis on the WTCCC (Affymetrix 6.0 data), HapMap3 CEU and TSI and EPICOLON populations.** A set of 15 000 independent markers was used to perform the analysis. The first eigenvector separates the Northern and Southern European populations.

Since there has been extensive literature on the differences amongst Northern and Southern European populations [25-28], we decided to perform a further PCA on 15,000 independent markers in order to compare allelic frequencies among the EPICOLON controls, the HapMap3 CEU and TSI populations, and the Wellcome Trust Case Control Consortium (WTCCC2) control cohorts [29]. This analysis effectively separated the Northern and Southern-European populations (Figure 2). Given this evidence, we decided to attempt replication of the best-associated markers (whether directly genotyped or imputed) at these 24 loci in an independent Spanish cohort (Phase II). Thirty-two SNPs were finally selected to be genotyped at this Phase II according to LD measures at the 24 loci and experimental design. The markers genotyped at each locus and their association values in the replication phase are described in Table 2.

**Table 2:** Association results for phase II and pooled analysis

**LOCUS**	**SNP**	**P PHASE II**	**OR (95% CI)**	**POOLED P**	**POOLED OR (95% CI)**
1p33	rs12080929	0.042	0.867 (0.722-0.994)	5.523E-03	0.866 (0.782-0.959)
1p33	rs12080061*	0.087	0.870 (0.743-1.020)	6.418E-05	0.793 (0.623-0.874)
2p25.2	rs4669394	0.736	1.038 (0.837-1.286)	6.693E-03	0.763 (0.627-0.928)
2p24.1	rs1554267	0.175	0.906 (0.785-1.045)	0.021	1.123 (1.018-1.240)
3p21.31	rs8180040	0.106	0.887 (0.767-1.026)	2.163E-06	0-784 (0-709-0-867)
3q12-q13	rs6438550	0.944	1.012 (0.731-1.400)	4.843E-03	0.723 (0.576-0.906)
5q35.1	rs11740081	0.508	0.935 (0.764-1.142)	4.772E-04	0.783 (0.683-0.898)
6q16.1	rs12213685	0.875	0.987 (0.842-1.157)	6.627E-03	1.220 (1.058-1.407)
6q16.1	rs4538713*	0.939	0.992 (0.804-1.224)	6.282E-04	0.702 (0.627-0.837)
6q23.1	rs12199765	0.231	1.108 (0.93-1.309)	2.693E-05	1.281 (1.141-1.438)
8p12	rs11996339	0.690	0.971 (0.842-1.120)	6.985E-05	0.817 (02739-0.903)
8p12	rs11987193	0.039	0.847 (0.724-0.992)	6.985E-05	0.786 (0.705-0.878)
8p12	rs12548021	0.234	1.095 (0.943-1.271)	2.587E-06	1.28 (1.155-1.418)
8q13.3	rs17788534	0.471	1.073 (0.885-1.301)	8.040E-04	1.249 (1.097-1.422)
8q22.1	rs3104964	0.081	1.139 (0.984-1.317)	4.239E-06	1.265 (1.144-1.398)
10p15.1	rs7074607	0.174	0.867 (0.705-1.065)	0.090	1.131 (0.981-1.305)
10q23.31	rs7087402	NA	NA	NA	NA
12q24.31	rs568489	0.701	1.022 (0.916-1.139)	1.197E-03	1.178 (1.067-1.300)
12q24.31	rs2686555	0.913	1.006 (0.903-1.121)	3.619E-03	1.158 (1.049-1.278)
13q32.3	rs17196583	0.270	0.903 (0.752-1.083)	0.0455	1.136 (1.003-1.288)
14q31.3	rs2057115	0.030	0.791 (0.639-0.977)	0.395	1.065 (0.921-1.231)
14q31.3	rs7148493	0.024	0.843 (0.727-0.977)	0.308	1.054 (0.923-1.165)
14q32.12	rs8177528	0.423	1.056 (0.910-1.225)	1.163E-03	1.183 (1.069-1.309)
15q21.3	rs4644804	0.528	1.037 (0.927-1.159)	0.011	1.139 (1.030-1.259)
15q25.3	rs16941001	0.484	1.067 (0.891-1.277)	5.457E-04	1.351 (1.139-1.603)
15q25.3	rs16941002*	0.560	1.055 (0.880-1.265)	0.127	1.143 (0.952-1.345)
17p13.2	rs16954697	0.280	1.119 (0.913-1.372)	3.168E-04	1.301 (1.127-1.501)
17p12	rs9898623	0.449	0.926 (0.757-1.131)	1.526E-03	0.734 (0.607-0.889)
18p11.22	rs10502376	0.986	0.973 (0.842-1.125)	0.011	0.880 (0.797-0.971)
18q21.2	rs2958182	0.714	1.001 (0.861-1.164)	1.831E-03	1.181 (1.064-1.311)
22q12.3	rs956119	0.369	0.897 (0.708-1.137)	5.911E-03	0.785 (0.660-0.933)
22q12.3	rs17725348*	0.273	0.875 (0.690-1.110)	3.976E-04	0.702 (0.629-0.895)

*Denotes imputed SNPs; NA: not available.

P-values and ORs for the SNPs genotyped in Phase II. All genomic positions correspond to NCBI36/hg18.

**Figure 3:**
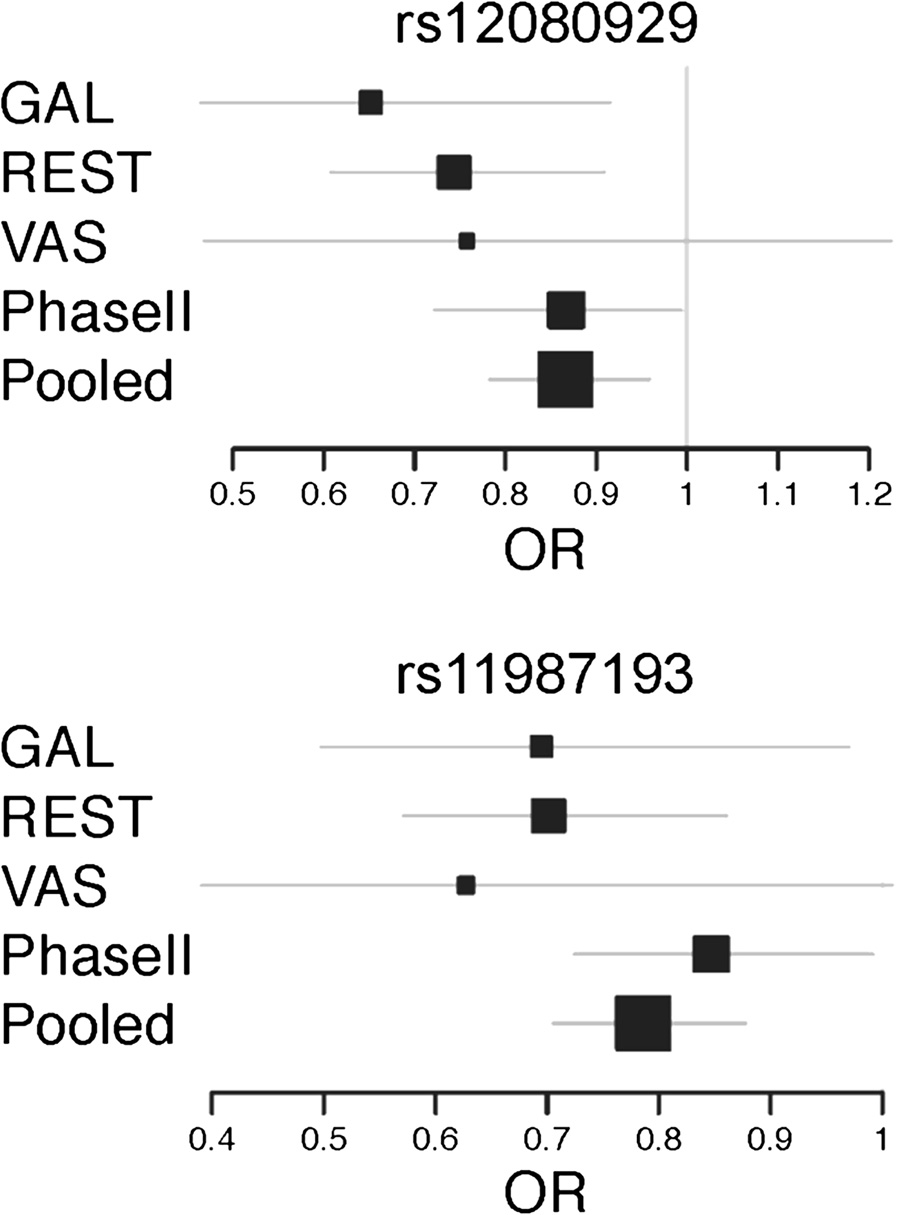
**Forest Plots for rs12080929 and rs11987193.** The figure represents the odds ratios and 95% confidence intervals for the two markers in all 3 populations subgroups from Phase I, the Phase II replication dataset and the pooled analysis of both Phases

Two out of the replicated 32 SNPs, rs12080929 (chromosome 1p33) and rs11987193 (chromosome 8p12) were successfully replicated at a nominal level of p<0.05 in this second Phase (P=0.044, OR=0.867 (0.722-0.994) and P=0.039, OR=0.847 (0.724-0.992), respectively). Although the association signals were very modest, pooled analysis of the data from both phases was consistent with the presence of a potential CRC susceptibility variant in these locations (pooled p values P=5.523x10^-3^ and P=6.985x10^-5^, respectively), and the signals remained significant across population subgroups (with the exception of the smallest VAS dataset) (Figure 3). Another two variants at locus 14q31.3 were significant in Phase II, but their OR were in different directions for each of the Phases, thereby reflecting a potential false positive event in Phase I. Given the marker sizes, this finding is entirely compatible with a random positive finding at a significance level of 0.05. With regards to phenotype analysis, rs12080929 seemed to be slightly overrepresented in males *vs.* females (P=0.042, OR=0.771 (0.600-0.991)), whereas rs11987193 was more prevalent in rectal cancers (P=0.028, OR=1.327 (1.031-1.707)). None of the other variables used in the subgroup analyses provided statistically significant results (Additional file 1: Table S2). It is also remarkable that none of the remaining three SNPs genotyped at these two loci (rs12080061 at 1p33, and rs11996339 and rs12548021 at 8p12) appeared to be replicated in this second phase, although rs12080061 showed a borderline p value in Phase II (P=0.087); in the case of rs11996339 and rs12548021 at 8p12 r-squared LD values among the three SNPs seem to show that the three markers are independent.

### Previous susceptibility loci

In addition to the search of new susceptibility variants, we also investigated the association signals for 19 of the known CRC susceptibility variants [3-5]. rs5934683 on Xp22.2 could not be evaluated due to the fact that sexual chromosome data need to be processed differently. Considering these markers were described from Illumina array tagSNP panels, most of them were not directly genotyped in our chip; therefore we proceeded with the evaluation of the association signals by considering the closest related proxy SNP (Table 3). Direct evidence of replication (taken as the presence of an associated SNP with one-tailed P<0.05 in the same LD block as the described tagSNP) was found for 2 of the SNPs (Table 3). The remaining loci, although not significant, showed ORs in the same directions as those described in the literature. We must however highlight that some of these SNPs (rs4444235 and rs1957636 at 14q22 and rs961253 andrs4813802 at 20p12) have already been found to present differences in Northern and Southern European populations, a fact that is consistent with our study not being able to replicate the association signals at these loci [28]. Imputation of the LD regions around these associated loci was conducted to search for an enhancing of the signals. No significant improvements were found, except for locus 15q13, for which an imputed SNP, rs16970016, 15kb upstream the *GREM1* gene, scored the best p value in our dataset (P=9.847x10^-5^). This SNP has a good pairwise relationship with the formerly described rs4779584 (r^2^=0.882) (data not shown).

**Table 3:** Replication results for the already-described loci

**SNP REF**	**LOCUS**	**REPORTED ALLELE**	**REPORTED MAF**	**ALLELIC OR (95% CI)**	**BEST PROXY AFFY 6.0**	**R2**	**OR (95% CI)**	**EPICOLON P value**
rs6687758	1q41	G	0.2	1.09 (1.06-1.12)	rs6691195	1	1.10 (0.92-1.33)	0.291
rs6691170	1q41	T	0.34	1.06 (1.03-1.09)	rs11579490	0.902	1.00 (0.86-1.17)	0.974
rs10936599	3q26	T	0.24	0.93 (0.91-0.96)	rs7621631	1	0.99 (0.83-1.19)	0.970
rs16892766	8q23	C	0.07	1.32 (1.21-1.44)	rs2437844	0.925	1.13 (0.87-1.46)	0.360
rs6983267	8q24	T	0.48	0.83 (0.79-0.87)	rs6983267	-	0.87 (0.75-1.01)	0.065
rs10795668	10p14	A	0.33	0.91 (0.86-0.96)	rs706771	0.896	0.89 (0.76-1.04)	0.15
**rs3802842**	11q23	C	0.29	1.21 (1.15-1.27)	rs3802840	1	1.19 (1.01-1.40)	**0.037**
rs11169552	12q13	T	0.26	0.92 (0.90-0.95)	rs11169544	1	0.99 (0.83-1.18)	0.891
rs7136702	12q13	T	0.35	1.06 (1.03-1.09)	rs7136702	-	1.14 (0.98-1.33)	0.0806
rs4444235	14q22	C	0.46	1.12 (1.07-1.18)	rs11623717	0.838	1.01 (0.88-1.17)	0.859
rs1957636	14q22	A	0.39	1.08 (1.06-1.11)	rs4901475	0.932	1.16 (0.99-1.34)	0.153
rs16969681	15q13	T	0.09	1.18 (1.11-1.25)	rs16969344	1	1.21 (0.96-1.53)	0.103
rs11632715	15q13	A	0.46	1.12 (1.08-1.16)	rs12592288	0.524	1.04 (0.81-1.12)	0.589
rs9929218	16q22	A	0.29	0.88 (0.83-0.92)	rs7186084	1	0.94 (0.80-1.10)	0.439
**rs4939827**	18q21	C	0.47	0.85 (0.81-0.89)	rs7226855	1	0.82 (0.71-0.95)	**8.204E-03**
rs10411210	19q13	T	0.10	0.79 (0.72-0.86)	rs7252505	0.831	0.90 (0.72-1.13)	0.363
rs961253	20p12	A	0.36	1.13 (1.08-1.19)	rs5005940	1	1.11 (0.92-1.26)	0.349
rs4813802	20p12	G	0.36	1.09 (1.06-1.12)	rs4813802	-	1.07 (0.91-1.25)	0.433
rs4925386	20q13	T	0.32	0.93 (0.91-0.95)	rs4925386	-	0.96 (0.82-1.12)	0.61

Association data for the 19 CRC risk variants (rs5934683 at Xp22.2 is not included). In bold two of the SNPs that showed direct evidences of association at a nominal p value<0.05.

## Discussion

Genome-wide association studies have so far successfully identified 20 CRC susceptibility SNPs [3-6]. Although this has been a significant improvement in the unravelling of the genetic basis of the disease, these variants alone do not completely explain all the inherited variation that has been attributed to CRC.

Following the lead of the previous studies, we addressed the issue of trying to detect new colorectal cancer susceptibility variants through the performance of a GWAS in a Spanish cohort. This was the first attempt to perform a CRC GWAS in a Southern European population. By these means, we were able to positively identify two new candidate variants that have shown good evidence of association with CRC risk: rs12080929 at 1p33 and rs11987193, at 8p12.

### Previous susceptibility loci

During the analysis, we were faced with the fact that, although there were no differences between case and control populations, there was a significant stratification issue determined by the hospital of origin of the samples. Because of this, the analyses had to be modified to match our case scenario without losing significant power. Nevertheless, the substructure in our cohort did not seem to greatly affect outcome quality. The evaluation on the 19 out of 20 already-described signals achieved direct replication for 2 of the loci (11q23 and 18q21). The other 17 markers did not show evidence of association, probably due to the lack of power in our cohort to detect such moderate effects. Nevertheless, OR directions were consistent with those previously published. We must highlight at this point that the best-associated markers for these regions did not always match with the best proxy for the already described SNPs. This would make sense if we consider that any given GWAS relies on an indirect approach, and we would expect the associated SNPs to only be tagging the real causative variant. Results for allele frequencies and ORs seem consistent with the bibliography [3-5]. We consider the replication of these loci an important achievement, since the majority of these association signals had not been previously evaluated in Southern European cohorts (with the notable exception of rs16892766 at 8q23.3, rs10795668 at 10p14, rs3802842 at 11q23, rs4779584 at 15q13, rs4444235 and rs1957636 at 14q22, and rs961253 and rs4813802 at 20p12 [5,28,30,31].

### Spanish GWAS results

The association analysis in itself provided with positive results for 24 different genomic loci at a p-value <0.0001. A first attempt at replication was aimed by inspection of these association signals on the British CORGI cohort [24]. However, none of the signals seemed to be shared between datasets. This lack of replication could be due to both false positive findings and artefacts from the calling algorithm, or to real differences between both populations leading to dissimilar abilities to tag the real causative variant [32]. The latter option has been recently proven to be possible, since differences in MAFs between Northern and Southern European populations have been described for SNPs rs4444235, rs1957636, rs961253 and rs4813802 [28].

A PCA analysis on the EPICOLON samples compared to the WTCCC control cohorts and the data from the HapMap3 CEU and TSI populations showed clear differentiation between the Northern and Southern European populations. Although not significant, SNP loadings also evidenced principal component 3 to be exclusively driven by a region of chromosome 8 (7.2-12Mb) where a common inversion is known to occur [33,34], whereas Eigenvectors 4-7 were driven by the HLA-A locus in the 6q21.2-21.3 region of chromosome 6, which has been also described as highly variable between populations [35]. Given this evidence, we proceeded on to replicate these 24 loci in an independent Spanish cohort.

SNPs rs12080929 and rs11987193 were successfully replicated in Phase II analyses. The former SNP, rs12080929 is located on an intronic position within hypothetical locus LOC388630 on 1p33. This predicted gene is believed to code for a single-pass type I membrane protein. Moreover, it lies 252kb upstream the *SLC5A9* gene, a member of the solute carrier family, which could be a feasible regulation target. SLC proteins constitute good candidates to harbour CRC susceptibility loci, since some family members have been proven to act as tumour suppressors. In fact, the *SLC5A8* gene is properly expressed in normal colon, but silenced in aberrant crypt foci through gene methylation [36].

The rs11987193 SNP is located in the 8p12 locus, 128kb downstream *DUSP4*. This gene is a member of the dual kinase phosphatase family, which are well-known tumour suppressors too [37]. They act through the downregulation of MAP kinases, thus preventing cellular proliferation and differentiation. Deletions in this gene have already been described to happen in other types of cancers, such as those of the breast and lung. In the case of CRC, *DUSP4* expression appears to be modulated by *KRAS* mutations [38-40]. Moreover, it has recently been described that DUSP4 expression is associated with microsatellite instability in CRC and causes increased cell proliferation [41].

The fact that these two SNPs were not replicated during the initial assessment of the association signals in the CORGI cohort, together with the North-South discrepancies seen in the PCA analysis, could be a sign of differences in the tagging of the real causative variant amongst populations. Even when Europeans are presumed to be genetically homogeneous, it is not unrealistic to believe that punctual LD variations may be actually happening within populations, and that these may constitute a certain impediment in our ability to replicate association signals [25,26].

Although none of the markers reached a final genome-wide significant p value, two of the SNPs in our study (rs12080929 and rs11987193) were favourably replicated in the second Phase and pooled analysis. However the limitations of this study (namely the modest sample sizes, further power restrictions also derived from the case-control age bias in Phase II and the potential differences between northern and southern European populations), we believe these two regions are good candidates for CRC susceptibility loci. The peculiarities of these loci, particularly those relating to potential Northern-Southern European differences, may have important repercussions on subsequent analysis. For this reason, the eventual identification of the functional variant is of uttermost importance. Finer mapping of the locus, coupled with additional replication efforts in larger cohorts will be needed to fully ascertain the relationship between these variants and disease. Moreover, it is important to highlight that, were these two SNPs true susceptibility variants, they would constitute a decrease in the CRC missing heritability fraction. This is an essential point in our road towards the identification of high-risk individuals within populations [7].

## Conclusions

The novelty of this study was the use of a Southern-European dataset to perform a CRC GWAS study that led to the identification of two new candidate variants: rs12080929 at 1p33 and rs11987193 at 8p12. These two SNPs are located near the *SLC5A9* and *DUSP4* loci, respectively. Some family members of the SLC proteins, as well as the *DUSP* gene, have been proven to act as tumour suppressors. Therefore, both of them could be good functional candidates for the association signals. Finer mapping and further replication in larger cohorts will be needed to ascertain their relationship with CRC susceptibility. The peculiarities of these loci, particularly those relating to potential Northern-Southern European differences, may have important repercussions on subsequent analyses.

## Competing interest

The authors declare that there are no conflicts of interest.

## Authors’ contributions

Conceived and designed the experiments: ACar, CR-P, SCB, ACas. Performed the experiments: CF-R, CP, AB-F, AA. Analyzed the data: CF-R, JB-C, IPMT, LC-C, CR-P. Contributed samples/analytical tools: JB-C, MJL, MB, LAL-F, LB, JC, DG, RX, MA, XB, RJ, XL, The EPICOLON Consortium, VM. Drafting the MS: CR-P, JBC, IPMT, SCB, ACar, ACas. Wrote the paper: CF-R. All authors read and approved the final manuscript.

## Supplementary Material

Additional file 1: Table S1 Phase I and Phase II cohorts. Main features and sample distribution of the phases. Gender count, hospital of origin and age statistics for cases and controls are shown for each phase.Table S2. Associations by phenotype. Phenotype counts and association values for SNPs rs12080929 and rs11987193 and each of the clinical variables studied.Table S3. Associated loci and imputation regions. Location of the 24 associated loci and description of the regions that were imputed for finer mapping. Click here for file

Additional file 2: Figure S1 Q-Q plots of p-value distribution. A: GAL ,B: REST; C: VAS; D:meta-analysis. Click here for file

Additional file 3: Figure S2 Imputation plots for the 24 loci associated with CRC in EPICOLON. P-value plots for the imputed markers in the associated regions. Diamonds represents typed SNPs, squares depict imputed markers, the biggest diamond is the best-associated SNP in the region, irrespective of typed/imputed status; red grading represents LD relationships . X axis: Chromosome location , Y axis: observed (-logP); Z axis: Recombination rate (cM/Mb). Click here for file

Additional file 4 Supplementary note. Members of the EPICOLON Consortium (Gastrointestinal Oncology Group of the Spanish Gastroenterological Association). Click here for file
