# Supplementary material for: A colorectal cancer genome-wide association study in a Spanish cohort identifies two variants associated with colorectal cancer risk at 1p33 and 8p12
Source: BMC Genomics. 2013 Jan 26;14:55. doi: 10.1186/1471-2164-14-55 (PMC3616862; doi:10.1186/1471-2164-14-55)
Supplement: Additional file 1: Table S1 — Phase I and Phase II cohorts. Main features and sample distribution of the phases. Gender count, hospital of origin and age statistics for cases and controls are shown for each phase.Table S2. Associations by phenotype. Phenotype counts and association values for SNPs rs12080929 and rs11987193 and each of the clinical variables studied.Table S3. Associated loci and imputation regions. Location of the 24 associated loci and description of the regions that were imputed for finer mapping. [file 1471-2164-14-55-S1.pdf]

Supplementary Table 1. Phase I and Phase II cohorts. Main features and sample distribution of the phases. Gender count, hospital of origin and age statistics for cases and controls are shown for each phase.

	**GENDER (MALE/FEMALE)**	**AGE MEAN (95% CI)**	**HOSPITAL OF COLLECTION/COHORT (number of samples)**
PHASE I: 881 CASES	550/332	71.2 (70.5-71.9)	Hospital Universitari Trias i Pujol (35)<br>Hospital del Mar (123)<br>Hospital Clinic (91)<br>Hospital General Universitario de Alicante (46)<br>Hospital Donostia (97)<br>Hospital Universitari Arnau de Vilanova (44)<br>Hospital Sant Pau (157)<br>Hospital do Meixoeiro (214)<br>Hospital de Calahorra (15)<br>Hospital Royo Villanova (22)<br>Hospital Universitario Central de Asturias (37)<br>Spanish National DNA bank (0)
PHASE I: 667 CONTROLS	392/275	65.7 (64.7-66.7)	Hospital Universitari Trias i Pujol (20)<br>Hospital del Mar (73)<br>Hospital Clinic (0)<br>Hospital General Universitario de Alicante (12)<br>Hospital Donostia (70)<br>Hospital Universitari Arnau de Vilanova (33)<br>Hospital Sant Pau (89)<br>Hospital do Meixoeiro (175)<br>Hospital de Calahorra (1)<br>Hospital Royo Villanova (0)<br>Hospital Universitario Central de Asturias (0)<br>Spanish National DNA bank (194)
PHASE II: 1436 CASES	875/561	69.6 (69.0-72.2)	Hospital Gregorio Maranon (104)<br>Hospital Sant Pau (125)<br>Catalan Institute of Oncology (439)<br>Complexo Hospitalario Universitario de Santiago (153) EPICOLON I (510)<br>Spanish National DNA bank (105)
PHASE II: 1780 CONTROLS	1068/712	52.0 (51.4-52.7)	EPICOLON I (450) Spanish National DNA bank (1330)

Supplementary Table 2. Associations by phenotype. Phenotype counts and association values for SNPs rs12080929 and rs11987193 and each of the clinical variables studied.

		**rs12080929**		**rs11987193**	
Phenotype	N1/N2/NA	OR (95%CI)	P	OR (95%CI)	P
Age at diagnosis (<60/>60)	132/657/3	1.181 (0.843-1.655)	0.332	1.134 (0.812-1.584)	0.460
MSI status (-/+)	509/59/224	1.072 (0.690-1.665)	0.758	1.020 (0.649-1.604)	0.913
Tumour location (colon/rectum)	542/233/17	1.000 (0.774-1.292)	0.998	1.327 (1.031-1.707)	0.028
(left/right)	533/242/17	0.855 (0.639-1.143)	0.290	1.001 (0.750-1.336)	0.996
Presence of previous adenomas (yes/no)	21/760/11	1.712 (0.703-4.166)	0.236	1.945 (0.755-5.011)	0.168
Family history of CRC (no/yes)	650/135/7	1.063 (0.801-1.411)	0.670	0.871 (0.651-1.164)	0.351
Sex (male/female)	481/309/2	0.771 (0.600-0.991)	0.042	0.932 (0.729-1.191)	0.573

Supplementary Table 3. Associated loci and imputation regions. Location of the 24 associated loci and description of the regions that were imputed for finer mapping.

CHR	LOCUS	IMPUTATION REGION
1	1p33	47,985,000-48,255,000
2	2p25.2	5,518,000-5,623,000
2	2p24.1	22,284,000-22,608,000
3	3p21.31	46,887,500-47,562,000
3	3q12-q13	120,991,750-121,488,000
5	5q35.1	172,706,000-172,719,000
6	6q16.1	99,253,000-99,325,000
6	6q23.1-q23.2	131,177,000-131,499,500
8	8p12	29,383,500-29,403,500
8	8q13.3	72,696,800-72,704,400
8	8q22.1	96,663,000-96,777,000
10	10p15.1	5,619,000-5,736,000
10	10q23.31	92,677,000-92,789,200
12	12q24.31	119,533,000-119,597,000
13	13q32.3	99,584,500-99,846,500
14	14q31.3	85,090,000-85,120,800
14	14q32.12	92,202,500-92,271,850
15	15q21.3	52,143,300-52,192,400
15	15q25.3	86,171,000-86,253,700
17	17p13.2	13,145,300-13,256,000
17	17p12	5,201,500-5,322,700
18	18p11.22	8,552,000-8,595,800
18	18q21.2	50,980,500-51,327,000
22	22q12.3	34,479,200-34,790,200
