# Supplementary figures and images for: A colorectal cancer genome-wide association study in a Spanish cohort identifies two variants associated with colorectal cancer risk at 1p33 and 8p12
Source: BMC Genomics. 2013 Jan 26;14:55. doi: 10.1186/1471-2164-14-55 (PMC3616862; doi:10.1186/1471-2164-14-55)

**Supplementary Figure 1. Q-Q plots of p-value distribution.** A: GAL; B: REST; C: VAS; D: meta-analysis.

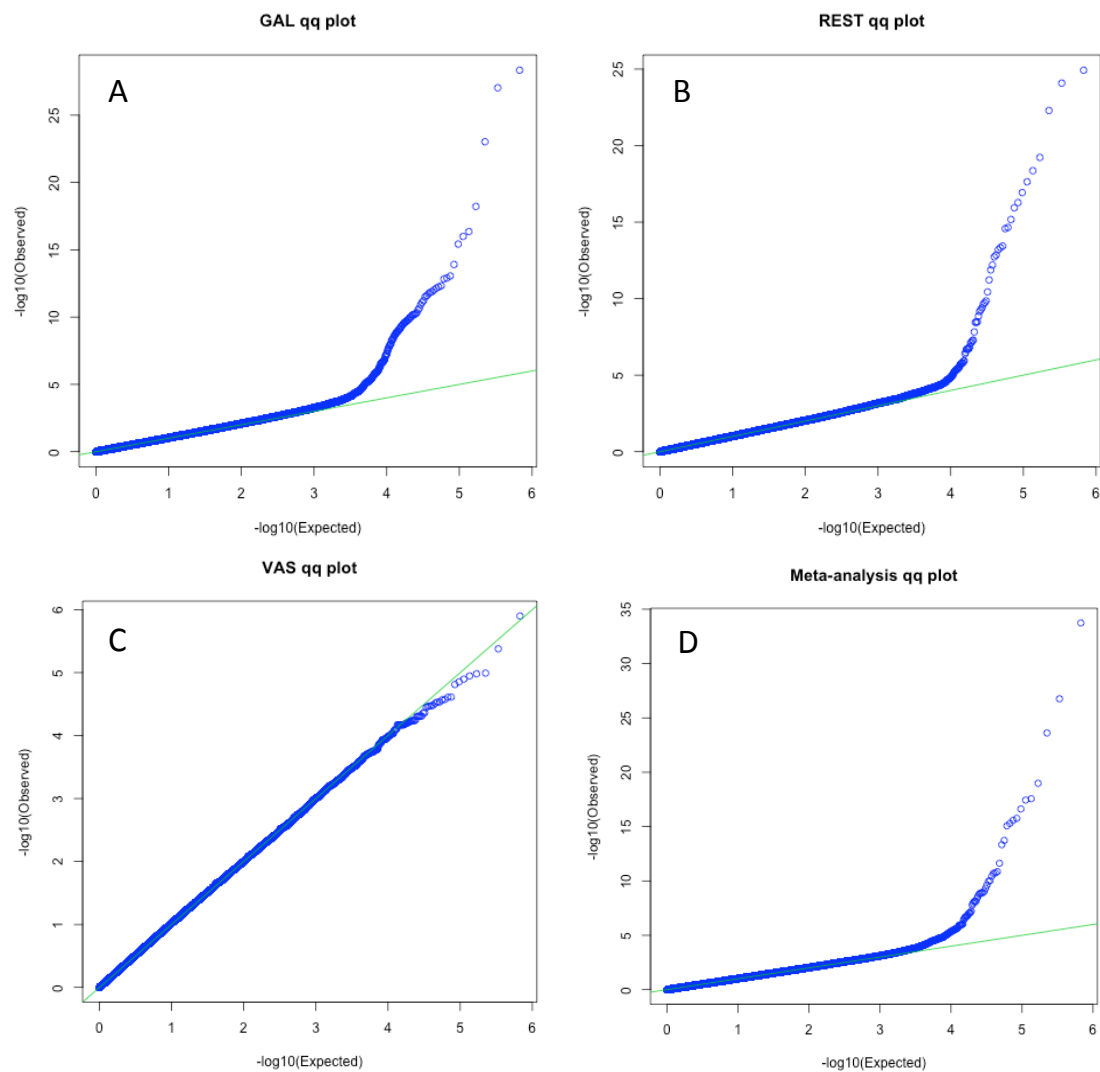

Supplement: Additional file 2: Figure S1 — Q-Q plots of p-value distribution. A: GAL ,B: REST; C: VAS; D:meta-analysis. [file 1471-2164-14-55-S2.pdf]
