# Supplementary material for: A colorectal cancer genome-wide association study in a Spanish cohort identifies two variants associated with colorectal cancer risk at 1p33 and 8p12
Source: BMC Genomics. 2013 Jan 26;14:55. doi: 10.1186/1471-2164-14-55 (PMC3616862; doi:10.1186/1471-2164-14-55)

Supplementary Figure 2. Imputation plots for the 24 loci associated with CRC in EPICOLON. P-value plots for the imputed markers in the associated regions. Diamonds represent typed SNPs, squares depict imputed markers; the biggest diamond is the best-associated SNP in the region, irrespective of typed/imputed status; red grading represents LD relationships. X axis: Chromosome location; Y axis: observed (-logP); Z axis: Recombination rate (cM/Mb).

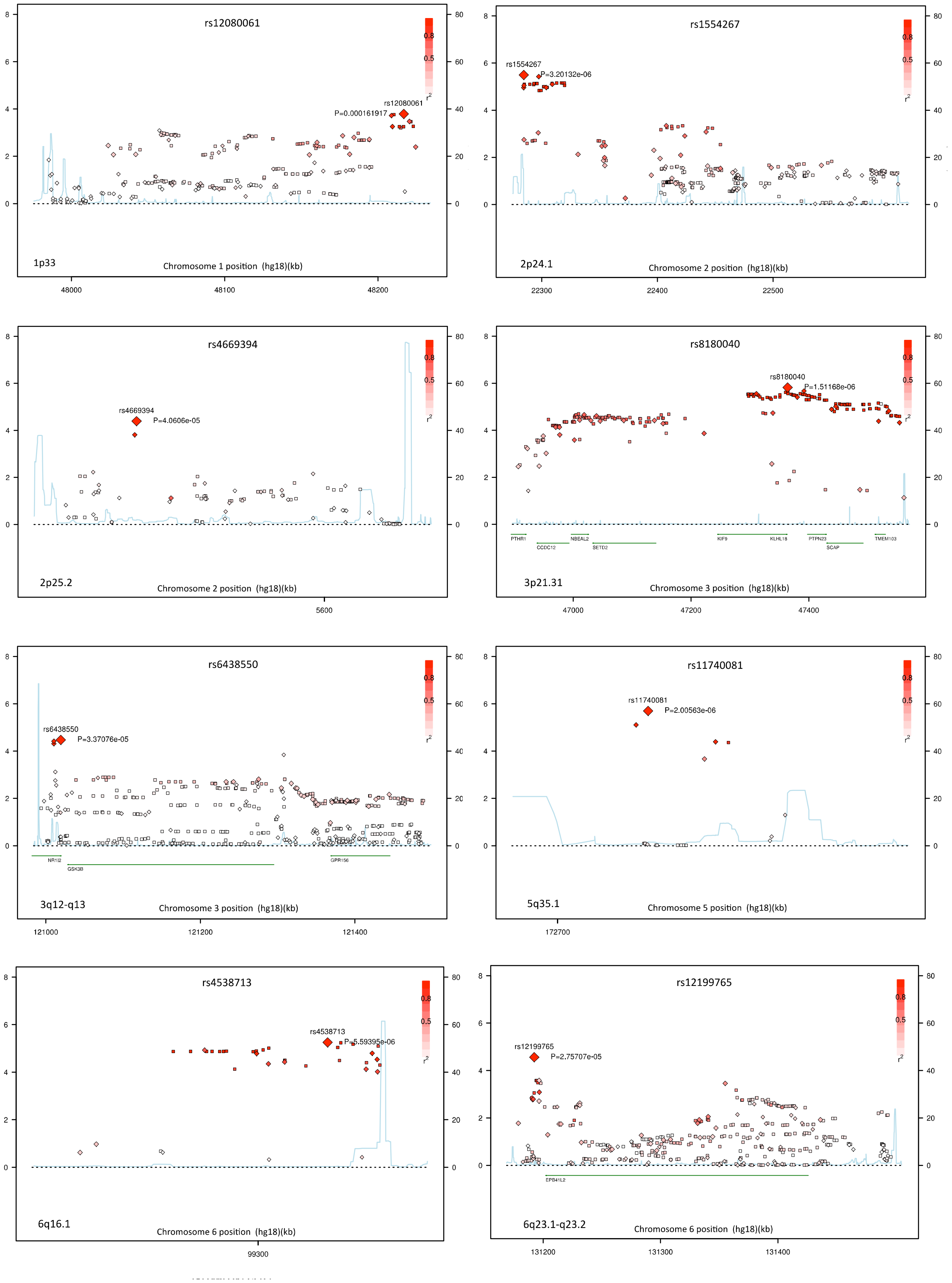

Supplementary Figure 2. (Continuation I).

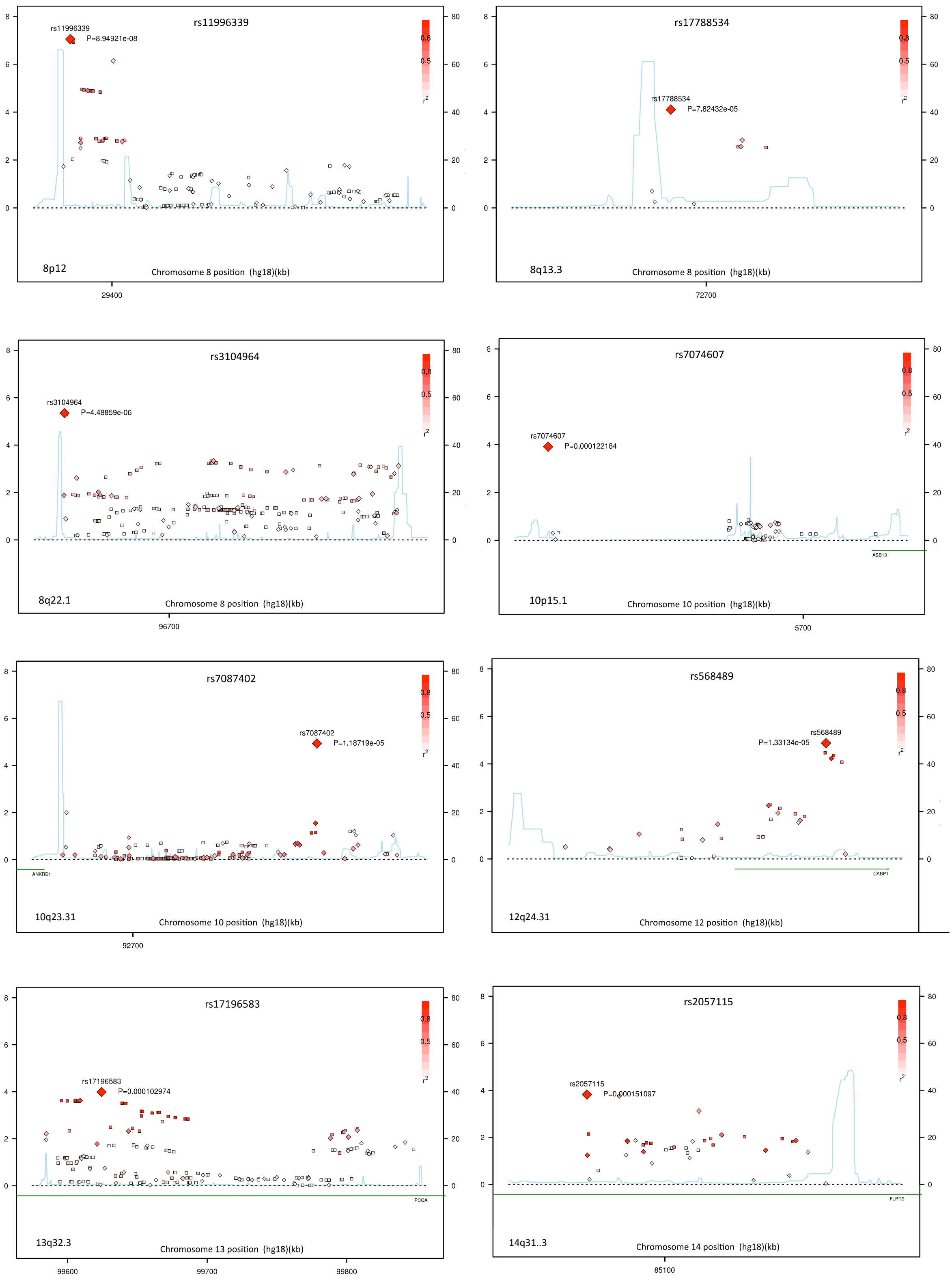

Supplementary Figure 2. (Continuation II).

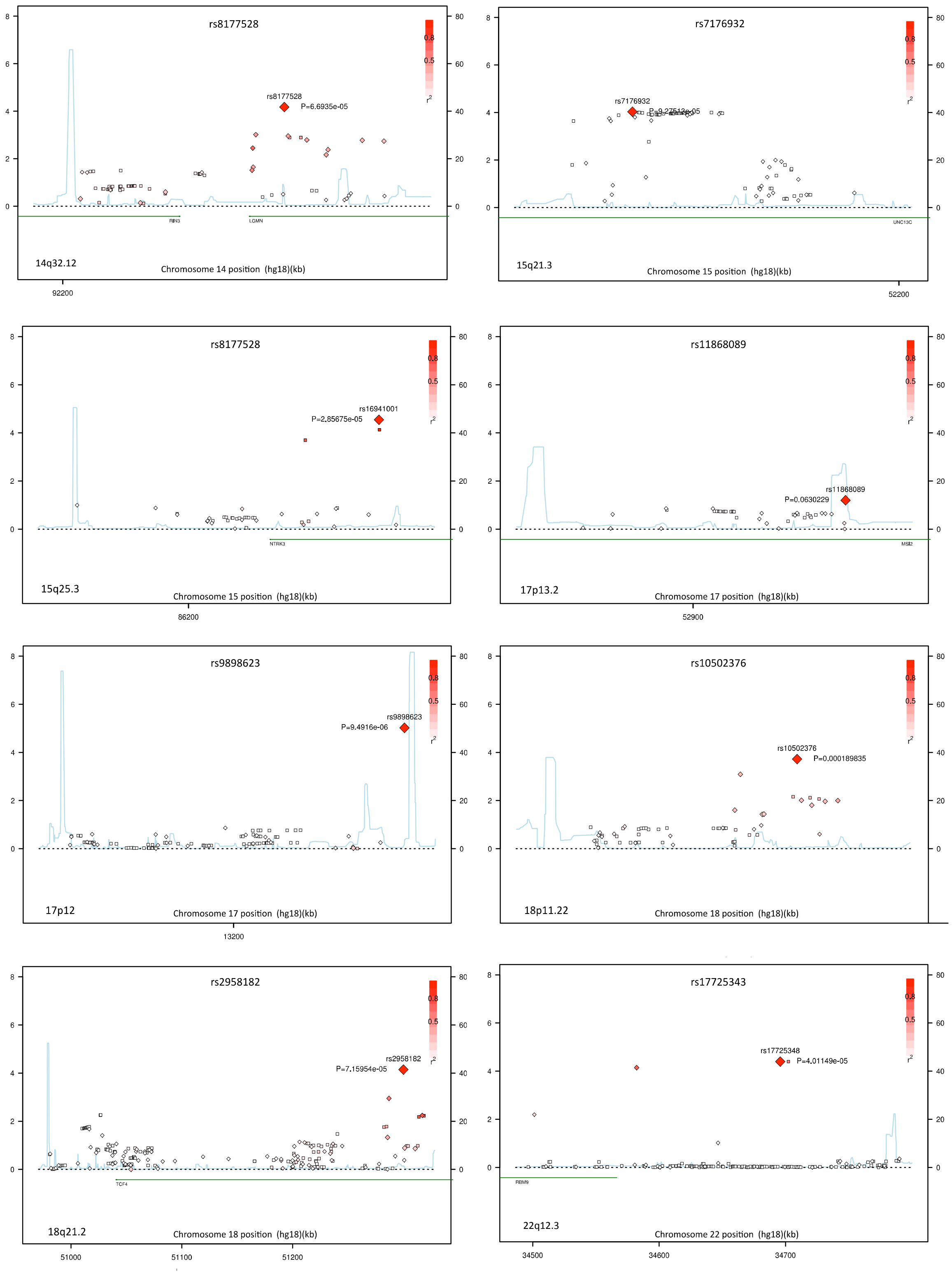

Supplement: Additional file 3: Figure S2 — Imputation plots for the 24 loci associated with CRC in EPICOLON. P-value plots for the imputed markers in the associated regions. Diamonds represents typed SNPs, squares depict imputed markers, the biggest diamond is the best-associated SNP in the region, irrespective of typed/imputed status; red grading represents LD relationships . X axis: Chromosome location , Y axis: observed (-logP); Z axis: Recombination rate (cM/Mb). [file 1471-2164-14-55-S3.pdf]
